# Hepatitis C Virus-Related Lymphomagenesis in a Mouse Model

**DOI:** 10.5402/2011/167501

**Published:** 2011-07-26

**Authors:** Kyoko Tsukiyama-Kohara, Satoshi Sekiguchi, Yuri Kasama, Nagla Elwy Salem, Keigo Machida, Michinori Kohara

**Affiliations:** ^1^Department of Experimental Phylaxiology, Faculty of Life Sciences, Kumamoto University, 1-1-1 Honjo, Kumamoto-shi, Kumamoto 860-8556, Japan; ^2^Department of Microbiology and Cell Biology, The Tokyo Metropolitan Institute of Medical Science, 2-1-6 Kamikitazawa, Setagaya-ku, Tokyo 156-8506, Japan; ^3^Department of Medical Virology, Faculty of Life Sciences, Kumamoto University, 1-1-1 Honjo, Kumamoto-shi, Kumamoto 860-8556, Japan; ^4^Clinical Pathology Department, Faculty of Medicine, Suez Canal University, Round Road Kilo 4.5, Ismailia, Egypt; ^5^Department of Molecular Microbiology and Immunology, University of Southern California, Keck School of Medicine, Los Angeles, CA 90033, USA

## Abstract

B cell non-Hodgkin lymphoma is a typical extrahepatic manifestation frequently associated with hepatitis C virus (HCV) infection. The mechanism by which HCV infection leads to lymphoproliferative disorder remains unclear. Our group established HCV transgenic mice that expressed the full HCV genome in B cells (RzCD19Cre mice). We observed a 25.0% incidence of diffuse large B cell non-Hodgkin lymphomas (22.2% in male and 29.6% in female mice) within 600 days of birth. Interestingly, RzCD19Cre mice with substantially elevated serum-soluble interleukin-2 receptor *α*-subunit (sIL-2R*α*) levels (>1000 pg/mL) developed B cell lymphomas. Another mouse model of lymphoproliferative disorder was established by persistent expression of HCV structural proteins through disruption of interferon regulatory factor-1 (*irf-1^_/_^*/CN2 mice). *Irf-1^_/_^*/CN2 mice showed extremely high incidences of lymphomas and lymphoproliferative disorders. Moreover, these mice showed increased levels of interleukin (IL)-2, IL-10, and Bcl-2 as well as increased Bcl-2 expression, which promoted oncogenic transformation of lymphocytes.

## 1. Introduction

The incidence of non-Hodgkin lymphoma (NHL) is rising worldwide and is higher in developed countries than in Africa and Asia [1]. B cell non-Hodgkin lymphoma is a typical extrahepatic manifestation frequently associated with hepatitis C virus (HCV) infection [2]. The prevalence of HCV infection in patients with B cell non-Hodgkin lymphoma is approximately 15% [3]. The HCV envelope protein E2 binds human CD81 [4], a tetraspanin expressed on various types of cells, including lymphocytes, and activates B cell proliferation [5]. Infection and replication of HCV were observed in B cells [6, 7] although the direct effects, particularly *in vivo*, have not been clarified. To determine the direct effect of HCV infection on B cells *in vivo*, we crossed transgenic mice with an integrated full-length HCV genome (Rz) under the conditional Cre/*lox*P expression system, with mice expressing the Cre enzyme [8] under transcriptional control of the B lineage-restricted gene *CD19 *[9]. 

To investigate the mechanism of development of lymphoproliferation or B cell non-Hodgkin lymphoma in HCV patients, we also developed a transgenic mouse model that conditionally expressed HCV cDNA (nucleotides 294–3435), including the viral genes that encode the core, E1, E2, and NS2 proteins, by using the Cre/*lox*P system (in core-NS2 [CN2] mice) [10, 11]. Conditional transgene activation of the HCV cDNA (core, E1, E2, and NS2) protects mice from Fas-mediated lethal acute liver failure, by inhibiting cytochrome *c* release from mitochondria [11]. Persistent HCV protein expression is established by targeted disruption of interferon regulatory factor-1 (*irf-1*), and high incidences of lymphoproliferative disorders are noted in *irf-1^−/−^* CN2 mice [12].

Previously, transgenic mice that expressed the HCV core protein were established using a promoter derived from hepatitis B virus [13], whereas mice that expressed structural or complete viral proteins were established using promoters derived from the albumin gene [14]. These mice were immunotolerant to the transgene and did not develop hepatic inflammation. However, they developed age-related hepatic steatosis and hepatocellular carcinomas. In contrast, the CN2 mice used in the present study were not immunotolerant to the *HCV* gene and developed hepatitis after the onset of HCV gene expression. However, the expression of *HCV* in these mice was usually lost after 21 days. Therefore, an animal model of persistent HCV protein expression is required for examining the effects of chronic HCV infection *in vivo*.

IFN signaling mediates tumor-suppressor effects and antiviral responses and is regulated by key transcription factors of the interferon-regulatory factor (IRF) protein family, including IRF1, IRF2, IRF3, IRF7, and IRF9. Targeted disruption of *irf-1* results in aberrant lymphocyte development and a marked reduction in the number of CD8^+^ T cells in the peripheral blood, spleen, and lymph nodes [15]. In addition, natural killer cell development is impaired in *irf-1^−/−^* mice [16]. The mechanisms by which HCV infection induces IFN resistance and influences the development of lymphomas are poorly understood. Therefore, we established an *irf-1^−/−^* CN2 mouse model of persistent HCV expression, which allowed us to investigate the effects of HCV on lymphatic tissue tumor development.

## 2. Spontaneous Development of B Cell Lymphomas in the RzCD19Cre Mouse

**Figure 1:**
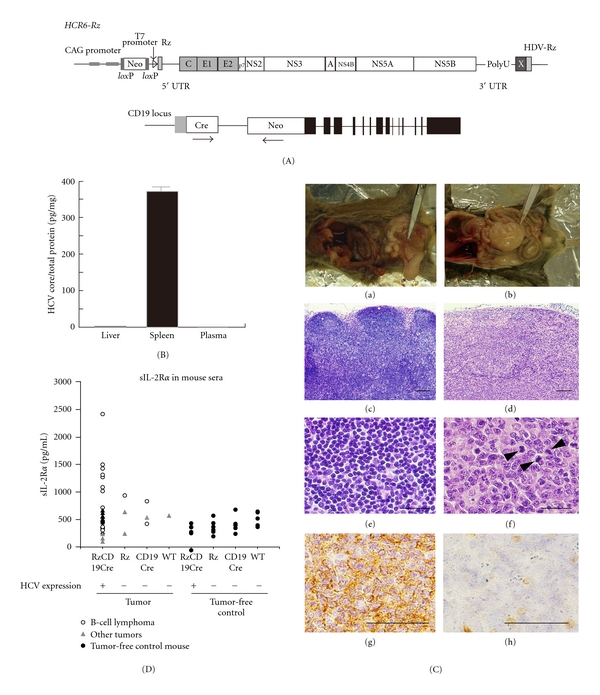
(A) The structure of the hepatitis C virus (HCV) transgene (HCR6-Rz); *HCV* gene expression was regulated by the Cre/*lox*P expression cassette (upper). The Cre transgene was located in the CD19 locus (bottom). (B) Expression of HCV core protein in the liver, spleen, and plasma of RzCD19Cre mice was quantified by core ELISA. Data represent the mean (SD) (*n* = 3). (C) Histological analysis of tissues from a normal mouse (a, c, e; lymph node from CD19Cre mouse; b, d, f) and B cell lymphoma (b, d, f; RzCD19Cre mouse). Paraformaldehyde-fixed and paraffin-embedded tumor tissues were stained with hematoxylin and eosin (H&E) (c–f); immunostaining of lymphoma with anti-CD45R (g) and anti-CD3 (h) is indicated. Scale bars, 100 *μ*m (c, d) and 20 *μ*m (g, h); arrow heads indicate mitotic cells. (D) Concentration of sIL-2R*α* in serum samples from tumor-free control mice, and RzCD19Cre and wild-type (WT) mice with or without B-cell lymphomas or other tumors.

The full-genome HCV expression was induced by the Cre/*lox*P system with CD19Cre (Figure 1(A)). Expression of HCV was mainly induced in B cells (Figure 1(B)). The incidence of B cell lymphomas in RzCD19Cre mice was 25.0% (22.2% in male and 29.6% in female mice) and was significantly higher than the incidence in the HCV-negative groups. Lymphomas were diagnosed as typical diffuse B cell non-Hodgkin lymphomas. Most were CD45R positive and located in the mesenteric lymph nodes (Figure 1(C)). Some were identified as intrahepatic lymphomas (incidence, 4.2%). *HCV* expression was detected in all B cell lymphomas of RzCD19Cre mice. Indeed, the expression of the *HCV* or HCV proteins induces the spontaneous development of B cell lymphomas, irrespective of the integrated site in the mouse genome.

Serum concentrations of IL-1*α*, IL-1*β*, IL-2, IL-3, IL-4, IL-5, IL-6, IL-9, IL-10, IL-12(p40), IL-12(p70), IL-13, IL-17, eotaxin, G-CSF, GM-CSF, IFN-*γ*, KC, MCP-1, MIP-1*α*, MIP-1*β*, RANTES, TNF-*α*, IL-15, FGF-basic, LIF, M-CSF, MIG, MIP-2, PDGF*β*, VEGF, alanine aminotransferase (ALT), and aspartate aminotransferase (AST) were not significantly different in the presence or absence of B cell lymphomas. Interestingly, the average sIL-2R*α* level was significantly higher in the sera from RzCD19Cre mice with B cell lymphomas (830.3 ± 533.0 pg/mL) than in that from tumor-free control groups, including the RzCD19Cre, Rz, CD19Cre, and wild-type (WT) mice (499.9 ± 110.2 pg/mL; *P* < 0.0057) (Figure 1(D)). The difference in the average sIL-2R*α* levels between the sera from groups with tumors other than B cell lymphomas (430.46 ± 141.15 pg/mL) and that from tumor-free control groups was insignificant (*P* > 0.05). Moreover, all RzCD19Cre mice with a relatively high level of sIL-2R*α* (>1000 pg/mL) presented with B cell lymphomas. A significant increase in sIL-2R*α* was also observed in MxCre/CN2-29 mice that expressed the HCV CN2 gene [8] and had B cell lymphomas, compared with tumor-free control (CN2-29) mice.

To examine whether sIL-2R*α* was derived from lymphoma tissues, we quantified IL-2R*α* concentrations in splenocytes, peripheral blood lymphocytes (PBLs), and B cell lymphoma tissues. The concentration of IL-2R*α* was significantly higher in splenocytes from RzCD19Cre mice than in splenocytes from CD19Cre mice. Moreover, the concentration of IL-2R*α* in B cell lymphoma tissues from RzCD19Cre mice was higher than that in splenocytes [8]. These results strongly suggest that B cell lymphomas directly contribute to the elevated serum concentrations of sIL-2R*α* in RzCD19Cre mice. They also strongly support the possibility that persistent expression of HCV could directly induce transformation of B cells.

## 3. Persistent HCV Expression and Lymphoproliferative Disorder

**Figure 2:**
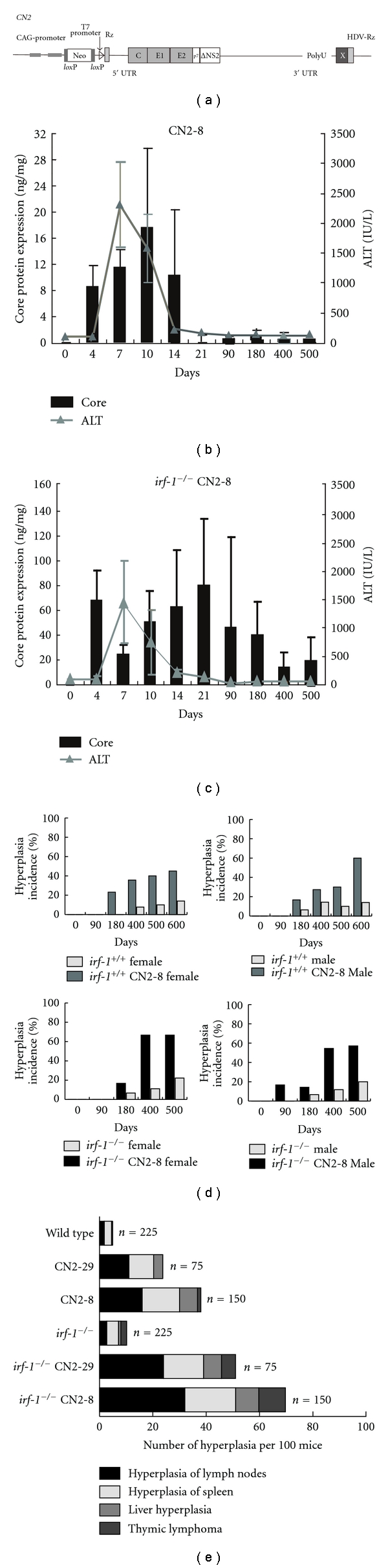
(a) The structure of the HCV transgene (core-NS2); gene expression was regulated by the Cre/*lox*P expression cassette. (b) and (c) Serum alanine aminotransferase (ALT) levels and core protein expression ELISA system in hepatocytes from CN2-8 (b) and *irf-1^−/−^* CN2-8 (c) mice after administration of AxCANCre (*n* = 225 for *irf-1^−/−^*, *n* = 75 for *irf-1^−/−^* CN2-29, *n* = 150 for *irf-1^−/−^* CN2-8, *n* = 225 for wild type, *n* = 75 for CN2-29, and *n* = 150 for CN2-8; total *n* = 900). (d) HCV protein expression enhanced hyperplasia in male and female CN2 and *irf-1^−/−^* CN2 mice. The occurrence of hyperplasia was monitored every 7 days for 600 days after administration of AxCANCre. (e) Histological analysis of spontaneous proliferative disturbances in CN2 transgenic mice. Of the 900 mice injected with AxCANCre, 25 of 75 (33%) CN2-29, 47 of 150 (31%) CN2-8, 29 of 75 (39%) *irf-1^−/−^* CN2-29, and 62 of 150 (41%) *irf-1^−/−^* CN2-8 mice developed proliferative disturbances.

RzCD19Cre mice are immunotolerant to HCV, because *HCV* is expressed in B cells before birth. In contrast, CN2 mice express *HCV *after they are administered the recombinant adenovirus that expresses the Cre enzyme (Figures 2(a) and 2(b)). The expression of HCV in these mice is usually lost after 21 days (Figure 2(b)) through removal of HCV-expressing hepatocytes by the immune response. To establish persistent HCV expression, we disrupted *irf-1* by crossing of RzCD19Cre mice with *irf-1^−/−^* mice. IRF-1 plays a significant role in the Th1-type immune response and its absence is expected to decrease the elimination of HCV-expressing cells. As expected, HCV expression in *irf-1^−/−^* CN2 mice persisted for more than 500 days (Figure 2(c)).

A significant percentage of the mice that expressed the HCV core protein (*irf-1^−/−^* CN2 mice) showed polyclonal lymphoid growth disturbances, including splenomegaly, expanded lymph nodes, adenocarcinoma in the abdomen or leg, and lymphoma of the liver or Peyer's patches (Figures 2(d) and 2(e)). In contrast, hepatocytes with abundant expression of HCV proteins rarely developed into hepatocellular carcinomas. Hematoxylin and eosin (H&E) staining of splenomegalic tissue showed extensive hyperplasia of the white pulp zones, in which the cortical zones contained lymphoid follicles and scattered germinal centers although mitotic figures were rarely observed. These results indicate that persistent expression of HCV proteins frequently induces lymphoproliferative disorders in addition to liver hyperplasia, which is consistent with the phenotype of patients with hepatocellular carcinoma.

The average ratio of T cells to B cells in the lymph nodes and spleens of CN2 mice was significantly higher than that in WT mice. The majority of CD3^+^ lymphocytes and a few CD8^+^ lymphocytes expressed CD4 on their surfaces. The proliferating cells were mainly CD4^+^ T cells although some were CD45R^+^B cells. The *irf-1^−/−^* CN2 mice also developed B cell lymphomas (data not shown). These results confirm that HCV protein expression induces lymphoproliferative disorders that involve excessive expansion of both T cells and B cells. The cell population that showed negative results for T cell receptor (*α*, *β*, *γ*, and *δ* isoforms) staining was smaller in *irf-1^−/−^* CN2 mice than that in the other mice.

The disruption of *irf-1* inhibited Fas-induced apoptosis, presumably by decreasing the levels of caspase-6 and caspase-7 messenger RNA. These results suggest that the reduced expression of effector caspases delays Fas-mediated apoptosis in *irf-1^−/−^* mice and prevents the elimination of HCV-expressing cells *in vivo*.

**Figure 3:**
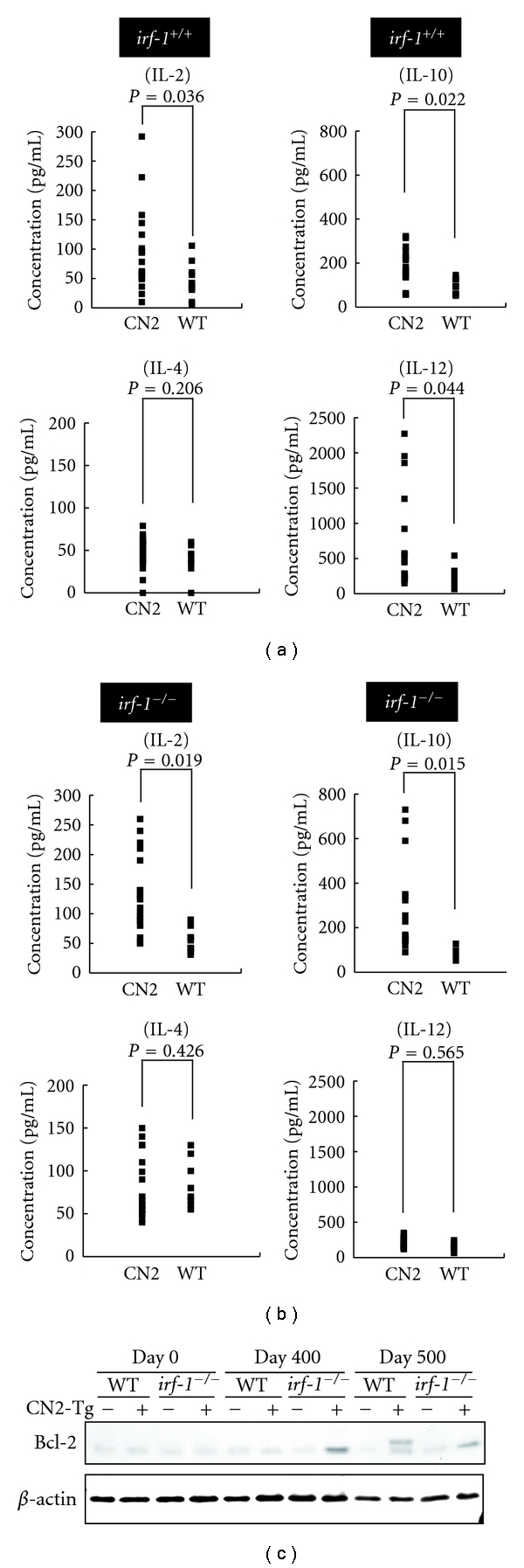
(a) Serum IL-2, IL-4, IL-10, and IL-12 levels in *irf-1^+/+^* CN2 (Tg+) and *irf-1^+/+^* WT mice measured by ELISA. *P* values <0.05 were considered significant. (b) Serum IL-2, IL-4, IL-10, and IL-12 levels in *irf-1^−/−^* CN2 (Tg+) and *irf-1^−/−^* WT mice measured by ELISA. *P* values are based on the mean cytokine concentrations. (c) Bcl-2 protein levels in the lymph nodes of *irf-1^+/+^* WT and *irf-1^−/−^* WT or transgenic (CN2-29) mice on day 0, day 400, and day 500 after administration of AxCANCre. Bcl-2 migrated at 26 kD.

The CN2 mice showed significantly increased levels of serum IL-2, IL-10, and IL-12 (Figure 3(a)). Notably, the CN2 mice with proliferative disturbances in the lymph nodes and spleen had dramatically elevated levels of these cytokines, suggesting that altered cytokine production is involved in aberrant lymphocyte proliferation or differentiation. In contrast, the *irf-1^−/−^* CN2 mice did not show elevated levels of serum IL-12, but had significantly higher levels of serum IL-2 and IL-10 than did *irf-1^−/−^* mice (Figure 3(b)). Thus, the disruption of *irf-1* negated the increase in the IL-12 level, but augmented the increases in the levels of IL-2 and IL-10 in CN2 mice. These results indicate that IL-2 and IL-10 play key roles in the induction of the lymphoproliferative phenotype in *irf-1^−/−^* CN2 mice. A significantly positive correlation was found between the cytokine levels and spleen weights of *CN2* gene-expressing mice with the *irf-1^+/+^* background (*R* = 0.43, *P* < 0.05, and *R* = 0.53, *P* < 0.05, resp.). These results indicate that IL-2 and IL-10 are involved in lymphoproliferation in viral protein-expressing mice.

Bcl-2 is an integral inner mitochondrial membrane protein, overexpression of which blocks the apoptotic death of pro-B-lymphocyte death [17]. *Bcl-2* transgene expression increases the oncogenic potential [18] and is linked with B cell neoplasm and t(14;18) translocation [19]. We therefore examined the level of Bcl-2 protein and found that it was upregulated in the lymph nodes of *irf-1^−/−^* CN2 mice after 400 days (Figure 3(c)).

IL-10 treatment in the presence of IL-2 greatly inhibited Fas-induced apoptosis in *irf-1^−/−^* CN2 mice compared with other groups (Table 1). Furthermore, *irf-1* disruption accelerated the resistance of splenocytes to Fas-induced apoptosis in the presence of IL-2, IL-10, and/or IL-12. In particular, IL-2 plus IL-10 treatment produced the strongest upregulation of the Bcl-2 mRNA levels in splenocytes of *irf-1^−/−^* CN2 mice. This indicates that IL-2, IL-10, and/or IL-12 contribute to upregulation of *bcl-2* expression, which subsequently inhibits Fas-induced apoptosis. Caspase-9 and caspase-3/7 activities were inversely correlated with the level of *bcl-2* expression. These results indicate that aberrant cytokine expression and disruption of IFN signaling affect *bcl-2* expression synergizing with HCV proteins, which is associated with the inhibition of caspase expression.

HCV core protein induced IL-2 and IL-10. Envelope protein E2 induced IL-12 expression. These results indicate that the HCV core and E2 proteins are responsible for IL-2, IL-10, and IL-12 expression. Core protein expression and IL-10 stimulation most strongly induced Bcl-2 expression (Table 2). From these results, core protein contributes significantly to the induction of Bcl-2 in the presence of cytokines. 

## 4. Conclusion

Our results show that the conditional expression of HCV proteins induces inflammation and lymphoproliferative disorders. Furthermore, established animal models will probably provide critical information for the elucidation of the molecular mechanism(s) underlying the spontaneous development of B cell non-Hodgkin lymphoma after HCV infection. The disruption of *irf-1* enhances lymphoproliferative disorders. Therefore, IRF-1-inducible genes probably play essential roles in suppressing HCV-induced lymphoma and in eliminating HCV protein-expressing cells. The overexpression of apoptosis-related proteins (including Bcl-2) and/or aberrant cytokine production are the primary events in HCV-induced lymphoproliferation.

The *HCV* gene has the potential to induce B cell lymphomas in RzCD19Cre mice, without inducing host immune responses against *HCV* gene product. This is in agreement with the results of a previous study, which indicate that viral elimination reduces the incidence of malignant lymphoma in patients infected with HCV [20]. The incidence of B cell lymphoma in the HCV transgenic mouse strain (MxCre/CN2-29) is high, and this strongly suggests that development of B cell lymphomas occurs via expression of the *HCV* transgene.

Recent findings indicate a link between sIL-2R*α* levels and hepatocellular carcinoma in Egyptian patients [21]. The level of IL-2R*α* was higher in splenocytes of RzCD19Cre mice than in those of CD19Cre mice; however, the differences in the serum concentrations of sIL-2R*α* between RzCD19Cre mice without B cell lymphomas and other control groups (Rz, CD19Cre, and WT) were insignificant. These results indicate that HCV increases IL-2R*α* expression in B cells; proteolytic cleavage of IL-2R*α* increased after B cell lymphoma development in the RzCD19Cre mice. The detailed mechanism by which HCV expression induces IL-2R*α* remains unclear, but HCV core protein induces IL-10 expression in mouse splenocytes [12]. IL-10 upregulates the expression of IL-2R*α* (Tac/CD25) in normal and leukemic B lymphocytes [22]. Therefore, through IL-10, the HCV core protein might induce IL-2R*α* in B cells of the RzCD19Cre mouse.

Disruption of *irf-1* enables the persistent expression of HCV protein. This leads to lymphoproliferative diseases resulting from reduced apoptosis (i.e., lower levels of caspase-1, caspase-6, and caspase-7 expression). HCV CN2 transgenic (Tg+) mice are resistant to Fas-induced apoptosis because of the inhibition of cytochrome *c* release from mitochondria [11]. Mice with disruption of *irf-1* have several defects in their innate and adaptive immunities, including lineage-specific defects in thymocyte development, and the development of immature T cells into mature CD4^+^ cells but not CD8^+^ T cells [16, 23]. IRF-1 controls the positive and negative selection of CD8^+^ thymocytes [24] and is required for the development of the Th1-type immune response. The absence of IRF-1 induces Th2-type immune response [16, 25]. The number of natural killer cells is dramatically reduced in *irf-1^−/−^* mice [16]. This defect may markedly increase viral protein expression and inhibit tumor surveillance mechanisms, leading to the development of non-Hodgkin lymphoma. Expression of the IL-12 p40 subunit is defective in *irf-1^−/−^* mice [16].

Hypermutation of the immunoglobulin genes in B cells induced by HCV infection is the cause of the lymphomagenesis observed in HCV infection [16, 26]. This finding may provide a more direct insight into lymphoma production, because HCV-induced hypermutation causes genetic instability and chromosomal aberrations, possibly resulting in neoplastic transformation [27]. In addition, the antiapoptotic phenotype resulting from sustained viral protein expression may enhance the survival of lymphocytes and inhibit activation-induced cell death to turn off the activated lymphocytes. The dysregulated cytokine profiles and sustained lymphocyte survival may alter the fates of regulatory T cells and dendritic cells [28].

In summary, the mouse model of B cell lymphoma and lymphoproliferative disorder represents a powerful tool to address the molecular mechanism of lymphoma development by HCV.

##  Conflict of Interests

The authors declare that there is no conflict of interests.

## Figures and Tables

**Table 1:** Synergistic effects of cytokines on Fas-induced apoptosis in *irf-1^−/−^* CN2 mice.

	None	IL-2 + IL-10	IL-2 + IL-12	IL-10 + IL-12
WT mice				

Bcl-2 fold increase	−	−	−	−
Annexin V + percentage	****	****	****	****
Caspase-9	**	**	**	**
Caspase-3/7	***	***	***	***

*irf-1^−/−^* mice				

Bcl-2 fold increase	−	−	−	−
Annexin V + percentage	***	***	***	***
Caspase-9	**	**	**	**
Caspase-3/7	**	**	**	**

CN2-29 mice				

Bcl-2 fold increase	−	+	−	−
Annexin V + percentage	***	**	***	***
Caspase-9	**	*	*	**
Caspase-3/7	**	*	*	*

*irf-1^−/−^* CN2-29 mice				

Bcl-2 fold increase	+	+++	++	++
Annexin V + percentage	**	−	*	*
Caspase-9	**	−	*	*
Caspase-3/7	*	−	*	*

Bcl-2: −, less than 2-fold increase; +, more than 2-fold increase; ++, more than 4-fold increase; +++, more than 6-fold increase (in comparison with mock treatment).

Annexin V: −, less than 20% decrease; *, up to 20% decrease; **, up to 40% decrease; ***, up to 60% decrease; ****, more than 60% decrease (in comparison with mock treatment).

Caspase-9: −, less than 10-fold decrease; *, up to 50-fold decrease; **, up to 200-fold decrease; ***, up to 400-fold decrease; ****, more than 400-fold decrease (in comparison with mock treatment).

Caspase-3/7: −, less than 200-fold decrease; ∗, up to 500-fold decrease; **, up to 1000-fold decrease; ***, more than 1000-fold decrease (in comparison with mock treatment).

**Table 2:** Synergistic effects of cytokines on Bcl-2 expression in WT and *irf-1^−/−^* mice in the presence of HCV core protein.

	WT mice	*irf-1^−/−^* mice
Mock	−	−
IL-2 + IL-10	+	+++
IL-2 + IL-12	+	++
IL-10 + IL-12	+	++
IL-2	−	−
IL-10	+	++
IL-12	−	−
IL-2 + Il-10 + IL-12	−	+

Bcl-2: −, less than 2-fold increase; +, more than 2-fold increase; ++, more than 4-fold increase; +++, more than 5-fold increase (in comparison with mock treatment).
